# Niches for Skeletal Stem Cells of Mesenchymal Origin

**DOI:** 10.3389/fcell.2020.00592

**Published:** 2020-07-10

**Authors:** Anastasiia D. Kurenkova, Ekaterina V. Medvedeva, Phillip T. Newton, Andrei S. Chagin

**Affiliations:** ^1^Institute for Regenerative Medicine, I.M. Sechenov First Moscow State Medical University (Sechenov University), Moscow, Russia; ^2^Department of Women’s and Children’s Health, Karolinska Institutet, Stockholm, Sweden; ^3^Department of Physiology and Pharmacology, Karolinska Institutet, Stockholm, Sweden

**Keywords:** skeletal stem cells, progenitors, osteoblasts, chondrocytes, MSCs, stem cell niche

## Abstract

With very few exceptions, all adult tissues in mammals are maintained and can be renewed by stem cells that self-renew and generate the committed progeny required. These functions are regulated by a specific and in many ways unique microenvironment in stem cell niches. In most cases disruption of an adult stem cell niche leads to depletion of stem cells, followed by impairment of the ability of the tissue in question to maintain its functions. The presence of stem cells, often referred to as mesenchymal stem cells (MSCs) or multipotent bone marrow stromal cells (BMSCs), in the adult skeleton has long been realized. In recent years there has been exceptional progress in identifying and characterizing BMSCs in terms of their capacity to generate specific types of skeletal cells *in vivo*. Such BMSCs are often referred to as skeletal stem cells (SSCs) or skeletal stem and progenitor cells (SSPCs), with the latter term being used throughout this review. SSPCs have been detected in the bone marrow, periosteum, and growth plate and characterized *in vivo* on the basis of various genetic markers (i.e., Nestin, Leptin receptor, Gremlin1, Cathepsin-K, etc.). However, the niches in which these cells reside have received less attention. Here, we summarize the current scientific literature on stem cell niches for the SSPCs identified so far and discuss potential factors and environmental cues of importance in these niches *in vivo*. In this context we focus on (i) articular cartilage, (ii) growth plate cartilage, (iii) periosteum, (iv) the adult endosteal compartment, and (v) the developing endosteal compartment, in that order.

## Introduction

### The Concept of a Stem Cell Niche

A stem cell niche is a dynamic and specialized microenvironment with a specific architecture that regulates self-renewal of stem cells, the balance between their quiescent and proliferative states, as well as their choice of fate and differentiation of their progeny. These functions are coordinated through communication between the stem cells and local environment, including neighboring cells, components of the extracellular matrix, and local gradients of morphogens and cytokines, in combination with physical factors such as oxygen tension, temperature, shear stress, etc. (Schofield, 1978; Wong et al., 2012; Ceafalan et al., 2018; Singh et al., 2019). Thus, to be comprehensive, studies on stem cells should take their appropriate niche into consideration as well. The several stem cell niches identified to date harbor hematopoietic (HSCs), neural, intestinal, epithelial, and muscle stem cells, among many others. Only some of these have so far been characterized extensively.

### The Hematopoietic Stem Cell (HSC) Niche Exemplifies the Complexity of These Microenvironments

The influence of the local environment on the behavior of the HSC niche (also called the bone marrow niche), which is among the most well-characterized, was first demonstrated by transplanting HSCs from intact to RARγ-deficient mice (Walkley et al., 2007). The HSC niche is now known to contain predominantly mesenchymal stromal and vascular endothelial cells and to receive signals from osteoblasts, macrophages, megakaryocytes, sympathetic nerve fibers and non-myelinated Schwann cells (Crane et al., 2017). The balance between differentiation of HSCs toward the myeloid or lymphoid lineage is regulated via signaling through the pathway involving delta-like canonical Notch ligand 4 (Dll4) in vascular endothelial cells (Tikhonova et al., 2019). Mesenchymal cells and their progeny control the size of this niche and, thus, the number of HSCs (Calvi et al., 2003; Zhang et al., 2003; Méndez-Ferrer et al., 2010) via the Notch, Bone Morphogenetic Protein (BMP) and Wnt signaling pathways (Calvi et al., 2003; Zhang et al., 2003; Fleming et al., 2008).

The major factors that promote homing of HSCs to their niche include angiogenin, stem cell factor (SCF, encoded by *Kitl*), stroma derived factor-1 (SDF1, also known as Cxcl12 and encoded by *Cxcl12*), angiopoetin, interleukin 7 (IL-7), Wnt5 and Dll4 (Calvi et al., 2003; Fleming et al., 2008; Crane et al., 2017). Recently, single-cell transcriptional profiling of bone marrow cells identified the cellular sources for these factors with a resolution higher than ever achieved previously: SCF, SDF1, angiogenin and membrane-bound Dll4 are produced by vascular endothelial cells; the highest levels of SDF1, SCF, and IL-7β are detected in mesenchymal cells; while Wnt5a is secreted by osteoblasts (Tikhonova et al., 2019). All these factors play a role in regulating the number of HSCs and their rate of renewal, as well as their mobilization and/or the direction in which they differentiate.

### Historical Perspective on Mesenchymal Stem Cells (MSCs) and Issues of Terminology

In addition to providing a home for HSCs, the skeleton gives fundamental structural support, facilitates locomotion and serves as an endocrine organ (DiGirolamo et al., 2012). Like all other adult tissues, skeletal tissues require a constant supply of cells for their renewal and maintenance throughout life. Friedenstein et al. (1968) identified a heterogeneous fibroblast-like cell population (colony-forming fibroblasts) in the bone marrow and spleen. In culture these colony-forming fibroblasts are capable of attaching to plastic, forming colonies and differentiating spontaneously into osteoblasts in culture; whereas when transplanted into guinea pigs they formed multiple skeletal tissues, such as bone, cartilage, muscle, and tendon (Friedenstein et al., 1970, 1974; Friedenstein, 1990).

This discovery remained largely unnoticed for two decades until Maureen Owen proposed that these colony-forming fibroblasts are bone marrow stromal stem cells (Owen and Friedenstein, 1988). Meanwhile, using limb bud mesenchymal cells from chick embryos, Arnold Caplan and colleagues were exploring mechanisms underlying osteo- and chondrogenic differentiation, coining the term mesenchymal stem cells (MSCs) for the first time (reviewed in Caplan, 1991). MSCs were reported to reside in the bone marrow, periosteum and muscle connective tissue, and could be expanded and differentiated into bone and cartilage both *in vitro* cultures, as well as in diffusion chambers implanted into mice (Caplan, 1991).

It should be emphasized here that the dogma in the 1980’s and early 1990’s was that the adult body only contained one type of stem cells, namely, hematopoietic stem cells. Accordingly, these initial discoveries of bone marrow stromal stem cells/MSCs were recognized and appreciated primarily by investigators interested in experimental hematology. However, this was changed by the publication by Pittenger (1999) of a protocol for the isolation, phenotypic characterization and expansion of human MSCs, which was well received in the atmosphere of excitement generated by the discovery of human embryonic stem cells.

Unfortunately, during subsequent decades the pronounced heterogeneity of MSCs, in combination with the wide variety of experimental approaches employed to isolate and culture these cells, led to confusion in this field. It also became clear that the term “mesenchymal stem cells” is inappropriate, since it does not reflect their properties accurately (Dominici et al., 2006; Bianco et al., 2008). Even Caplan, the inventor of this term, made pleas that it be changed (Caplan, 2010, 2017). In 2006 the International Society of Cellular Therapies recommended the terminology “multipotent mesenchymal stromal cells” instead, defining these as clonogenic, multipotent, self-renewing cells that express CD105, CD73, and CD90, but not CD45, CD34, CD14, CD11b, CD79α, or HLA-DR, and are capable of osteogenic, chondrogenic and adipogenic differentiation (Dominici et al., 2006). Nonetheless, the term MSCs is utilized so widely by researchers around the globe that it is unclear when, or even if this terminology will be clarified, an issue that continues being discussed (Bianco and Robey, 2015; Caplan, 2017; Ambrosi et al., 2019).

In the present review we focus almost exclusively on *in vivo* characterization of MSCs, which are often referred to as skeletal stem cells (SSCs) (Bianco and Robey, 2015; Ambrosi et al., 2019). Since in many cases these cell populations are characterized on the basis of genetic markers which actually label heterogenous populations (Debnath et al., 2018; Tikhonova et al., 2019), below we will use the term skeletal stem and progenitor cells (SSPCs).

In recent years several types of SSPCs at different locations within the skeleton and with different functions and markers have been described (Sacchetti et al., 2007; Méndez-Ferrer et al., 2010; Ding et al., 2012; Greenbaum et al., 2013; Zhou B.O. et al., 2014; Chan et al., 2015; Li et al., 2017; Mizuhashi et al., 2018, 2019; Newton et al., 2019). However, our understanding of the local microenvironment in which these various SSPCs reside and the factors involved in regulating their behavior is still evolving. Below, on the basis of what is known to date, we make some suggestions concerning the nature of each particular niche. We have arranged our comments in the order of the following anatomical locations: articular cartilage, epiphyseal cartilage, periosteum, adult endosteal compartment and developing endosteal compartment.

## SSPCs in the Articular Cartilage and their Maintenance

The superficial zone of articular cartilage contains chondroprogenitors capable of generating chondrocytes, both *ex vivo* (Dowthwaite et al., 2004) and *in vivo* (Kozhemyakina et al., 2015) and also capable of reconstituting the entire articular cartilage (i.e., the middle and deep zone chondrocytes) in postnatal mice (Li et al., 2017). These cells have the following characteristics:

(i) Expression of several markers commonly utilized for the identification of SSPCs (BMSCs//MSCs), including CD105, vascular cell adhesion protein 1 (Vcam1, also known as CD106), CD166, Notch1, Stro, Dkk3, Tenascin C, Erg, CD73, CD34 and smooth muscle actin (Dowthwaite et al., 2004; Jiang and Tuan, 2015; Kozhemyakina et al., 2015; Li et al., 2017).
(ii) The ability to form colonies and differentiate into chondrocytes, osteoblasts and adipocytes *in vitro* (Alsalameh et al., 2004; Dowthwaite et al., 2004; Jiang and Tuan, 2015).
(iii) A cell cycle that is slower than that of their progeny (Li et al., 2017).

Kozhemyakina et al. (2015) showed that superficial cells expressing proteoglycan 4 (Prg4, also called lubricin) *in vivo* give rise to articular chondrocytes while themselves remaining at the surface of the articular cartilage for at least one year, suggesting that they are capable of renewal. To address this question directly, we utilized these same Prg4-CreERT2 transgenic mice in combination with clonal analysis *in vivo* and found that, indeed, these superficial cells can divide asymmetrically, generating one daughter cell that remains at the surface while the other undergoes further differentiation (Li et al., 2017). We have also observed symmetrical division of these superficial cells, following which both daughter cells remain at the cartilage surface, continue to express stem cell markers and exhibit characteristic flat morphology (Li et al., 2017). These observations indicate renewal of chondroprogenitors and, therefore, we refer to these superficial Prg4+ chondroprogenitors as articular SSPCs (art-SSPCs).

The microenvironment in which art-SSPCs reside is quite unique – there is no vascularization, no innervation, the cells are exposed to the synovial fluid and, in connection with locomotion, are frequently subjected to mechanical stress. Surgical translocation of hypertrophic chondrocytes from the growth plate to the articular surface leads to their gradual adoption of a phenotype resembling that of art-SSPCs (Chau, 2014), lending support to the concept that this unique microenvironment promotes art-SSPC properties.

Synovial fluid is enriched in lubricin, a large proteoglycan product of the *Prg4* gene and hyaluronic acid, which, together with phosphatidylcholine, form a stable boundary layer that minimizes the shear stress associated with locomotion (Seror et al., 2015). Both this layer and other molecular components of the synovial fluid might be involved in creating the appropriate microenvironment. In addition, synovial fluid transports oxygen and nutrients, along with soluble factors secreted by the art-SSPCs, chondrocytes and cells of the synovial membrane, all of which might also play a role in creation of the microenvironment required for the maintenance and renewal of art-SSPCs.

In connection with movements, joints are regularly subjected to mechanical forces and mechanical stimuli influence the fates of various types of stem cells (Engler et al., 2006; Guilak et al., 2009). Although the principal component of stress might be similar for art-SSPCs and underlying chondrocytes, shear stress is obviously higher at the surface, where art-SSPCs are located. Indeed, mechanical loading and shear stress might actually be part of the microenvironment required by art-SSPCs.

This possibility is supported by the finding that fluid flow shear stress enhances the expression of *Prg4* by art-SSPCs through a mechanism dependent on prostaglandin-endoperoxide synthase 2 (Cox2) and cAMP response element-binding protein (CREB) (Ogawa et al., 2014) and perhaps also on Wnt/β-catenin (Xuan et al., 2019). Furthermore, mechanical stress may elevate expression of Gremlin-1 (an antagonist of BMP signaling) by cells in the middle zone (Chang et al., 2019). Intra-articular injection of recombinant Gremlin-1 promotes the development of osteoarthritis, and blocking this factor improves healing (Chang et al., 2019). BMPs are involved in the restoration of cartilage destroyed by osteoarthritis, apparently by stimulating the proliferation and differentiation of chondrocytes (Deng et al., 2018). BMP7 is produced primarily by the cells of the superficial zone (Chubinskaya et al., 2000), and if this process is inhibited, less aggrecan is produced (Söder et al., 2005).

Further support for an impact of mechanical stress on art-SSPCs is provided by the finding that in connection with destruction of articular cartilage by immobilizing a joint, cells at the cartilage surface (the location of art-SSPCs) are lost first (Hagiwara et al., 2009; Correa Maldonado et al., 2013). However, this observation must be interpreted with care, since immobilization not only reduces mechanical stress, but also limits circulation of the synovial fluid, potentially impairing respiration by the cells in cartilage and limiting their access to nutrients.

In contrast to the underlying chondrocytes located in an extracellular matrix rich in type II collagen and negatively charged proteoglycans, the extracellular matrix surrounding art-SSPCs is rich in type XXII collagen and thrombospondin 4 (*Thbs4*) and contains only low amounts of proteoglycans (Gray et al., 2001; Feng et al., 2019). Gene array analysis has revealed that the art-SSPCs themselves exhibit low expression of typical components of the cartilage matrix, including aggrecan (*Acan*), collagen II (*Col2a1*), collagen IX (*Col9a1*), collagen XI (*Col11a1*), and matrilin-1 (*Matn1*), while expressing tenascin C (*Tnc*) and CD44 (*Cd44*) (Yasuhara et al., 2011) at high levels. Thus, art-SSPCs are surrounded by an extracellular matrix that differs substantially in composition from the one surrounding chondrocytes. Since the extracellular matrix plays a role in regulating the behavior of SSPCs (Engler et al., 2006; Guilak et al., 2009; Ferreira et al., 2018), this unique composition may be part of the specific microenvironment required for the maintenance and renewal of art-SSPCs.

Due to their unique location, art-SSPCs are not in direct contact with any other types of cells and even become gradually separated from their progeny by abundant extracellular matrix. Nevertheless, committed progeny and cells of the synovial membrane are probably the only cells capable of communicating with art-SSPCs, although communication with macrophages and other blood cells that infiltrate into the synovial space cannot be excluded, especially in connection with pathological processes.

As described for various niches in which epithelial or hematopoietic stem cells reside, interplay between stem cells and their progeny is common (Ferraro et al., 2010). Such interplay between mesenchymal cells is exemplified by the niche in the mouse incisor, where committed progeny signal back to the stem cells via interaction between delta like non-canonical Notch ligand 1 (Dlk1) and Notch (Walker et al., 2019). In mouse articular cartilage Notch ligands and receptors are expressed in a distinct spatiotemporal pattern, with *Notch1* being expressed by neonatal art-SSPCs (Hayes et al., 2003; Dowthwaite et al., 2004). With the exception of the superficial zone, other Notch receptors (Notch 2, 3, and 4) and ligands (Jagged 1 and 2) are distributed throughout the articular cartilage (Hayes et al., 2003).

Attenuation of Notch with DAPT, an inhibitor of gamma-secretase, prevents the proliferation of chondroprogenitors, thereby depleting the number of progeny cells and leading to a region poor in cells beneath the superficial zone (Dowthwaite et al., 2004). The depletion of Notch signaling in the entire adult articular cartilage employing Aggrecan-CreERT transgenic mice results in progressive degeneration of the extracellular matrix, including loss of proteoglycan, along with fibrosis in the articular cartilage and altered chondrocyte morphology (Liu et al., 2016). Thus, as in the mouse incisor (Walker et al., 2019), the Notch signaling pathway may participate in regulating mouse art-SSPCs. At the same time, it is worth mentioning that Notch1 has been detected immunohistochemical in all zones of human articular cartilage (Ustunel et al., 2008; Grogan et al., 2009) or, in one case, only during osteoarthritis (Mahjoub et al., 2012), but that the existence of putative human art-SSPCs remains to be established.

In addition to Notch, art-SSPCs express members of other signaling pathways that have been evolutionarily conserved and play crucial roles during development, including members of the Wnt family, BMPs, and members of the family of transforming growth factors, their receptors and modulators (Yasuhara et al., 2011; Grogan et al., 2013). Wnt/β-catenin signaling has been detected in the superficial zone of the articular cartilage of adult mice, but not in the chondrocytes of the middle and deep zones (Xuan et al., 2019). Using transgenic mice mutated specifically with respect to cartilage (i.e., collagen type II- or type XI-driven transcripts), Yasuhara et al. (2011) found that activation of Wnt/β-catenin signaling enhances the number of slowly dividing art-SSPCs, whereas deletion of β-catenin stimulates chondrogenic differentiation and induces complete loss of art-SSPCs (Yasuhara et al., 2011). Mice in which β-catenin has been knocked-out specifically in the superficial zone develop osteoarthritis early than controls (8 weeks after surgical induction of this condition), in combination with reduced expression of lubricin and destruction of the superficial zone (Xuan et al., 2019). In contrast, Prg4-Cre-driven stabilization of β-catenin enhances resistance against osteoarthritis, as well as expression of the *Prg4* gene in the superficial zone of articular cartilage (Xuan et al., 2019).

Analysis of the gene expression profiles revealed that the expression of both canonical and non-canonical Wnt ligands (*Wnt2b, Wnt4, Wnt5a, Wnt11*, and *Wnt16*) by art-SSPCs differs significantly from that by chondrocytes (Yasuhara et al., 2011). The relatively high levels of mRNA encoding the non-canonical Wnt ligands *Wnt5a, Wnt5b, Wnt9a*, and *Wnt16* in art-SSPCs were later confirmed (Xuan et al., 2019). Treatment of cultured art-SSPCs with non-canonical Wnt5a or Wnt5b elevated their expression of *Prg4*, but also caused canonical responses, i.e., elevations in the expression of *Ctnnb1* (β-catenin-coding gene) and *Axin2* genes (Xuan et al., 2019). Activation of the canonical β-catenin pathway with Wnt3a elevates the number of art-SSPCs during their first two passages in culture (Yasuhara et al., 2011), although inhibitors of this pathway pose no effect in these *in vitro* experiments (Yasuhara et al., 2011). Altogether, findings both *in vivo* and *in vitro* indicate that β-catenin signaling in the superficial zone contributes to the homeostasis and maintenance of the art-SSPCs phenotype in articular cartilage, but the exact nature of its involvement remains to be further elucidated.

**FIGURE 1:**
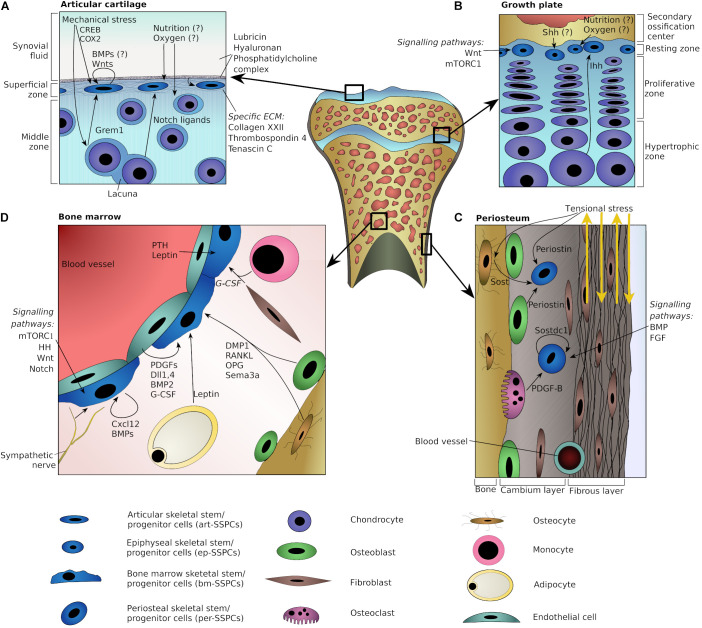
Schematic representation of plausible niche components at various skeletal locations. Potential niche components are shown for articular cartilage **(A)**, epiphyseal growth plate **(B)**, periosteum **(C)**, and adult bone marrow **(D)**. Arrows indicate directions of interactions or point toward specific cells. Question marks indicate potential players with the lack of strong direct evidences.

Altogether, the microenvironment supporting the maintenance of art-SSPCs and the generation of articular chondrocytes probably involves signaling from underlying chondrocytes (e.g., through Notch ligands), a unique set of proteins in the extracellular matrix (e.g., collagen type XXII and thrombospondin-4) and mechanical stress (Figure 1A).

## The Stem Cell Niche in the Epiphyseal Growth Plate

Recently, it was shown that the resting zone of the postnatal epiphyseal growth plate of mice harbors slowly cycling stem cells that express parathyroid hormone-related protein (PTHrP) and are capable of generating epiphyseal chondrocytes (Mizuhashi et al., 2018). These stem cells can be detected a year after labeling at 6 days of postnatal age. Here, to emphasize their location, we will refer to them as epiphyseal skeletal stem progenitor cells (ep-SSPCs).

*In vitro* these ep-SSPCs can directly differentiate into osteoblasts, chondrocytes and, to a lesser extent, adipocytes (Mizuhashi et al., 2018); whereas *in vivo* they generate chondrocytes, which then undergo hypertrophy and transdifferentiate into osteoblasts and stromal cells (Mizuhashi et al., 2018). During the juvenile period of growth, additional ep-SSPCs probably move from the perichondrium into the periphery of the resting zone, as revealed by genetic tracing of Axin2-positive perichondrial cells (Usami et al., 2019).

One key feature that distinguishes stem cells from their progenitors is their ability to maintain their own population and, at the same time, generate committed progeny via asymmetric cell division (Post and Clevers, 2019). This implies that clones of stem cells are stable, whereas those of their progenitor cells are only temporary. Indeed, labeling of all chondrocytes within the growth plate of mice revealed the formation of stable clones between 3 and 4 weeks (Newton et al., 2019). This coincides with the maturation of the secondary ossification center (SOC), which may constitute a part of the stem cell niche responsible for the renewal of ep-SSPCs (Newton et al., 2019). In line with this observation, tracing of PTHrP+ cells in mice from 6 days of age revealed that temporary clones are formed initially, whereas the formation of stable clones is observed only after 25 days of age (Mizuhashi et al., 2018).

Thus, PTHrP+ cells within the growth plate may act as slowly cycling chondroprogenitors that acquire the ability to self-renew only upon formation of the niche. This proposal is supported by the phenotypic and functional changes that these cells undergo at the time of niche formation, i.e., expression of stem cell markers such as CD73 and CD49e, among other changes in their transcriptional profile (Newton et al., 2019), as well as development of differential responsiveness to activation of hedgehog (HH) signaling (based on comparison of Mizuhashi et al., 2018 and Newton et al., 2019; see also below). Interestingly, in line with earlier indications (Ferraro et al., 2010), these findings suggest that the niche not only maintains stem cells, but also primes their phenotypic and functional properties.

The stem cell niche in the growth plate, which we refer to as the epiphyseal stem cell niche (Chagin and Newton, 2019), is probably formed by the SOC when it has matured so that bone cells and extracellular matrix is close to the ep-SSPCs (Newton et al., 2019). The variety of cells present in the SOC include osteocytes, osteoblasts, stromal cells, mesenchymal cells, and hematopoietic cells of the bone marrow, as well as nerve fibers and the cells that form blood vessels. Furthermore, appearance of the SOC alters local mechanical loading (Xie et al., 2019a) and, since the blood vessels from the SOC are near the avascular cartilage, probably nutrition and oxygenation as well.

Thus, the number of potential actors in connection with formation of the epiphyseal niche is quite high. We found that several types of cells in the SOC produce Sonic hedgehog (Shh) (Newton et al., 2019), a diffusible morphogen that can participate in the formation of a stem cell niche (Lai et al., 2017). In theory, Shh could diffuse from the SOC to the resting zone of the growth plate to help achieve this formation. Indeed, pharmacological inhibition of HH signaling results in fusion of the growth plate (Kimura et al., 2008; Newton et al., 2019). At the same time, the Indian hedgehog (Ihh) protein, another member of hedgehog family, is expressed by pre-hypertrophic and hypertrophic chondrocytes and then diffuses through the growth plate to stimulate the expression of PTHrP (encoded by *Pthlh*) by chondroprogenitors (Vortkamp et al., 1996). It is important to stress that specific genetic ablation of Ihh within the growth plate induces premature fusion (Maeda et al., 2007).

Moreover, activation of HH through either systemic or intra-articular injection of smoothened agonist (SAG) promotes the proliferation of ep-SSPCs (Newton et al., 2019). Interestingly, SAG has this effect only after formation of the SOC, actually inhibiting the expansion of ep-SSPCs if injected earlier (Mizuhashi et al., 2018). Altogether, hedgehog signaling clearly plays major roles in the epiphyseal stem cell niche, such as sustaining renewal of the stem cells, but the detailed role of each hedgehog ligand in the niche remains to be elucidated.

It is important to emphasize that at the same time as pre-hypertrophic chondrocytes are an important source of Ihh, they are also descendants of the ep-SSPCs. Thus, it can be said that the descendants of the ep-SSPCs participate in the formation of the stem cell niche. In this context it is worth mentioning that ablation of parathyroid hormone receptor 1 (PTHR1, encoded by *Pthr1*), which brings the region in which Ihh is expressed and the ep-SSPCs closer together, triggers apoptosis in slowly dividing (label-retaining) chondrocytes in the resting zone of 4-week old mice (Chagin et al., 2014). However, upon niche formation there is very little overlap between these label-retaining and the column-forming cells, which thus may appear to be different populations (Newton et al., 2019).

The observation that HH influences ep-SSPCs in different ways before and after the onset of the niche indicates that their response is dependent on the present state of their microenvironment. Accordingly, these cells may also respond to other morphogens and signaling in a different manner before and after the onset of the niche. If so, this will complicate use of the results of many genetic manipulations (which are often performed on fetal/neonatal growth plate, long before the onset of the niche) in unraveling the mechanisms underlying juvenile growth.

In connection with these mechanisms, the Wnt/β-catenin pathway affects slowly cycling cells in the resting zone. Specifically, transient activation of this pathway in the growth plate of 2–3-week-old mice results in fusion of this structure 7 weeks later (Yuasa et al., 2009). Furthermore, excessive β-catenin signaling causes hypertrophic chondrocytes to undergo apoptosis (Yuasa et al., 2009), potentially an indirect mechanism of action on ep-SSPCs. On the other hand, ablation of β-catenin from the entire growth plate of 4–6-day-old mice employing Col2-CreERT causes loss of the label-retaining cells from the resting zone at 4 weeks of age (Candela et al., 2014). At the same time, a deficiency in β-catenin does not impair the size and functions of hypertrophic cells, but rather their transdifferentiation into osteoblasts (Houben et al., 2016). These observations, together with the well-established role of the β-catenin pathway in other stem cell niches (Clevers and Nusse, 2012), indicate that β-catenin may well play a key role in the epiphyseal stem cell niche, although direct evidence for this is still lacking.

The mammalian target of rapamycin complex 1 (mTORC1), another signaling pathway that has been conserved throughout evolution, may also be involved in regulating the behavior of ep-SSPCs in the epiphyseal niche. Activation of this pathway, by ablation of the tuberous sclerosis complex 1 (*Tsc1*) gene, shifts the division of colony-forming cells within the growth plate from symmetric to asymmetric; whereas inactivation by ablation of the regulatory associated protein of mTORC1 (Raptor) (encoded by the *Rptor* gene) leads to the loss of the clones within the growth plate (Newton et al., 2018, 2019). In these experiments genetic ablation was performed in 3-day-old mice, and although no phenotype in the growth plate is observed until the SOC is formed (Newton et al., 2018, 2019) indirect effects cannot be excluded entirely. Altogether, it appears likely that mTORC1 signaling regulates the balance between symmetric and asymmetric division of ep-SSPCs.

In summary, components of the SOC and gradients of the two HH morphogens Shh and Ihh appear to play important roles in the epiphyseal stem cell niche. In addition, mTORC1 and Wnt/β-catenin signaling pathways are likely to be involved in the regulation of ep-SSPCs (Figure 1B).

## The Periosteal Stem Cell Niche

The periosteum is a thin membrane covering the external surface of numerous skeletal elements. In the case of long bones, this membrane is formed in connection with embryonic bone formation, when the core of the mesenchymal condensation differentiates into chondrocytes (forming a cartilaginous skeletal element) and the surrounding layer of mesenchymal cells forms a tight membrane-like structure with cells of characteristic flat morphology called the perichondrium. When mineralized bone starts to form at the expense of the cartilage anlagen, this perichondrium lining newly formed bone is re-designated as the periosteum, while the lining around cartilage is still called the perichondrium (Dwek, 2010).

The presence of SSPCs in the perichondrium/periosteum was indicated by surgical observations already 250 years ago. In 1779 Michele Troja observed the osteogenic capacity of the periosteum and Ollier (1867), confirmed that the inner layer of the isolated periosteum (cambium) can produce bone and is essential for transversal bone growth. Since these early discoveries, the presence of stem/progenitor cells in the periosteum has been confirmed and described in greater detail in numerous clinical observations (Lazzeri et al., 2009). Moreover, Caplan and colleagues (Nakahara et al., 1990a, b, 1991a,b) have shown that cells in the periosteum possess chondro- and osteogenic capacity *in vitro*.

The genetic identification and detailed characterization of periosteum *in vivo* was not performed until later, including by recent lineage tracing strategies in mice using a variety of drivers. Tracing of α-smooth muscle actin (α-SMA)-positive cells revealed their periosteal location, as well as their osteo- and chondrogenic capacities and contribution to the healing of bone fractures (Grcevic et al., 2012). However, it turned out that the α-SMA promoter is active in several different cell types (Yuan, 2015; Alarcon-Martinez et al., 2018; Ge et al., 2018), making this initial observation ambiguous.

Subsequently, the finding that Cathepsin K (*Ctsk* gene), generally employed as a marker for osteoclasts, can also be used to label periosteal cells allowed detailed characterization of the cellular hierarchy within the periosteum, including identification of CtsK+CD200+ cells as periosteal stem cells (here per-SSPCs) (Debnath et al., 2018). Under normal physiological conditions in the adult periosteum, per-SSPCs differentiate into cells of the osteoblast lineage (Debnath et al., 2018), whereas during healing of a non-stabilized fracture, these cells also differentiate into chondrocytes (Colnot, 2009; Colnot et al., 2012; Ortinau et al., 2019). Interestingly, single-cell analysis of CtsK-traced cells from both the perichondrium and periosteum of 6-day-old pups revealed four different subpopulations with: (i) expression of the α-SMA gene *Acta2* at high levels, (ii) high levels of Sca1 (encoded by *Ly6a*), (iii) expression of the chondrocyte markers *Col2a1* and *Sox9*, and (iv) expression of *Bglap* and *Alpl*, markers for osteoblasts. Finally, a subpopulation of α-SMA+ cells also expressing MX dynamin-like GTPase 1 (Mx1) was recently shown to behave like per-SSPCs, contributing to cells of the osteoblast lineage, participating in the healing of fractures, and restoring their own populating following serial transplantations (Ortinau et al., 2019).

At present it is not entirely clear to what degree these populations of CtsK+ and Mx1+α-SMA+ cells overlap. During neonatal life, CtsK+ cells probably contain a subpopulation of α-SMA+ cells (subpopulation 4, Figure 3h in Debnath et al., 2018), but the situation in the adult periosteum is more complicated: CtsK+ cells do not express the leptin receptor (LepR) or CD140a, whereas Mx1+α-SMA+ cells do (Debnath et al., 2018; Ortinau et al., 2019). Nonetheless, in both of these studies the per-SSPCs were found to be located close to the surface of bone, potentially in their own stem cell niche.

The robustness of the periosteum is provided by its collagen, while the elasticity of this structure is based on fibers of elastin reinforced by collagenous Sharpey’s fibers anchored to the bone at an angle of 45°. This structural arrangement maintains the periosteal tissue in a state of anisotropic tensional pre-stress (Knothe Tate et al., 2016). Changes in this stress following bone fracture and periosteal lesion leads to deformation of the structural collagen fibrils and cellular nuclei, as well as rapid emigration of cells (Yu et al., 2017).

Such observations have led to the proposal that the periosteal tissue acts as a mechanosensor and it is assumed that the normal state of stress promotes quiescence in the periosteal stem cell niche (Knothe Tate et al., 2016; Yu et al., 2017). Theoretically, this quiescence could be achieved through the action of different proteins such as periostin (*Postn*) on per-SSPCs. Indeed, using immunofluorescence periostin+ cells have been detected in the inner layer of the periosteum located along the cortex of bone, but not in the bone marrow or endosteum (Duchamp de Lageneste et al., 2018). Furthermore, after fracture, expression of periostin in the periosteum is elevated and, in addition, if this protein is knocked out, bone regeneration and callus size and quality are all reduced (Duchamp de Lageneste et al., 2018). Transplantation of wild-type periosteal cells into periostin-KO mice improve fracture healing in these animals to a remarkable degree (Duchamp de Lageneste et al., 2018). Thus, both mechanical forces and proteins of the extracellular matrix appear likely to be involved in the formation of a stem cell niche for per-SSPCs.

In response to mechanical stimulation the levels of both periostin mRNA and protein in total-bone extracts increase, an effect mediated by a reduction in expression of the sclerostin (*Sost*) gene by osteocytes (Bonnet et al., 2009). Interestingly, through interaction with the integrin αVβ3 receptor, periostin inhibits *Sost* expression directly (Bonnet et al., 2012). In a similar manner, periostin is required for the anabolic effects of parathyroid hormone (PTH) on cortical bone (Bonnet et al., 2012).

A paralog of *Sost*, the Sclerostin domain-containing protein 1 (*Sostdc1*, also known by S*ost-Like, Wise, Ectodin* and *Usag-1*) is expressed primarily in the periosteum (Collette et al., 2016) and contributes to limb morphogenesis (Collette et al., 2013). Sostdc1 has been described as an antagonist of BMP signaling (Yanagita, 2005) and both Sostdc1 and Sost inhibit Wnt signaling via lipoprotein receptor-related proteins (LRP) co-receptors (Li et al., 2005; Semenov et al., 2005). The cells in the periosteum that express Sostdc1 also express Nestin (encoded by *Nes*) and α-SMA, suggesting that these are osteochondral progenitor cells which participate actively in callus formation during bone repair (Collette et al., 2016). Absence of Sostdc1 hastens the expansion and differentiation of a subpopulation of per-SSPCs during bone healing (Collette et al., 2016). Such findings indicate that both Sostdc1 and periostin promote and maintain the quiescence of per-SSPCs in the periosteal niche, either directly and/or by modulating Wnt/β-catenin signaling.

Healing of bone fractures involves a well-orchestrated series of biological events, including hematoma, inflammation, revascularization, bone formation and remodeling (Bahney et al., 2019). Interestingly, a subpopulation of per-SSPCs that express both Mx1 and α-SMA (Mx1+α-SMA+ per-SSPCs) migrate toward the site of injury *in vivo* (Ortinau et al., 2019). These cells, which express the chemokine receptors *Ccr5* and *Ccr3* on their surface, migrate toward the injury along a gradient of Ccl5 (encoded by *Ccl5, Rantes*), a ligand for these receptors (Ortinau et al., 2019). In response to inflammation Ccl5 is synthesized by a number of different cells, including CD8+ T-cells, NK-cells, macrophages, epithelial cells, fibroblasts and platelets. Following bone injury in both *Ccl5*- and *Ccr5*-deficient mice, formation of new bone is impaired and the volume of the external callus reduced, indicating the importance of this migratory mechanism for regeneration (Ortinau et al., 2019) and suggesting that per-SSPCs or their immediate progeny must leave their niche in order to take part in bone regeneration.

In addition to the markers described above, per-SSPCs were recently reported in the outer layer of the periosteum of mice and found to express Nestin during the early postnatal period as well as being positive for LepR tracing in adulthood (Gao et al., 2019). Deficiency in cytokine colony stimulating factor 1 (CSF-1) in mononuclear cells, macrophages and osteoclasts leads to a significant reduction in the numbers of osteoprogenitor cells expressing Nestin, Osterix (Osx, also known as Sp7 transcription factor) as well as LepR-traced cells, possibly indicating that cells of the macrophage lineage play an important role in supporting periosteal niches (Gao et al., 2019). In addition, platelet-derived growth factor subunit B (PDGF-B), secreted by tartrate-resistant acid phosphatase (TRAP)-positive cells, stimulates the expression of *Postn* by cells derived from the periosteum. Furthermore, ablation of the receptor for PDGF-B, PDGFRβ, in cells of the LepR-lineage impairs their recruitment to the bone surface, as well as the formation of new bone tissue both during active bone growth and bone repair (Gao et al., 2019).

Although there is evidence that BMP, Fibroblast Growth Factor (FGF), hedgehog and Notch signaling are all involved in bone regeneration mediated by the periosteum, their direct involvement in regulation of per-SSPCs not yet been demonstrated. Ablation of *Bmp2* prior to fracture (utilizing the ubiquitous R26-CreERT strain coupled with *Bmp2* floxed mice) eliminates formation of the callus, with only undifferentiated cells being observed at the site of the injury (Wang et al., 2011). Insertion of a bone graft from *Bmp2*-deficient mice into the wild-type host fails to rescue differentiation, but administration of exogenous BMP2 partially improved healing (Wang et al., 2011). At the same time, the level of expression of *Bmp4* and *Bmp7* in mice deficient in *Bmp2* does not change in response to bone fracture (Tsuji et al., 2006). These observations indicate that BMP2 has an important role to play in connection with the healing of bone fractures, but whether this factor regulates the behavior of per-SSPCs directly remains to be determined.

The role of the FGF family of proteins in stimulating osteogenesis in connection with fracture healing is well established (Charoenlarp et al., 2017). Although *Fgf4, 8* and *20* are not expressed in the callus, expression of *Fgf2, 9, 16*, and *18* in this structure is elevated temporarily in response to bone fracture, a response important for the healing process (Schmid et al., 2009). At the same time, expression of the FGF receptor 1 (*Fgfr1*) by periosteal osteoprogenitor cells is enhanced in response to fracture (Nakajima et al., 2001), suggesting that FGF proteins may be involved in the regulation of osteogenesis by per-SSPCs.

Following bone damage, expression of the Notch ligands Jagged1 (*Jag1*) *Notch2* and *Hes1* in the callus rises with time (Dishowitz et al., 2012). Ablation of Jagged1 (*Jag1*) or inhibition of canonical Notch signaling in osteochondral progenitors by the dominant-negative mastermind-like (dnMAML) protein in the paired related homeobox 1 (Prx1)-Cre mouse strain causes periosteal expansion of cortical bone, while simultaneously lowering the expression of osteogenic genes (Youngstrom et al., 2016).

*Ihh* is expressed by pre-hypertrophic chondrocytes in the callus (Le et al., 2001) and may also be involved in the healing of fractures by periosteal cells. *In vitro* activation of HH signaling promotes the osteo- and chondrogenic differentiation of periosteal mesenchymal cells (Wang et al., 2010). Deletion of Smoothened (frizzled class receptor gene *Smo*), an intracellular member of the HH pathway, utilizing ubiquitous R26-CreER mice attenuates the recovery of bone mass after fracture and diminishes cell proliferation in the callus (Wang et al., 2010).

In summary, the periosteal niche may encompass its unique extracellular matrix (of which periostin is an important component), mechanosensing mechanisms, and signaling cues from cells of the osteo- and macrophage lineages. The signaling molecules likely to participate in regulation of the per-SSPCs include Sost, Sostdc1, and PDGFs, as well as members of the BMP and FGF families. Inflammatory chemokines, and in particular Ccl5, may cause per-SSPCs to leave their niche (Figure 1C).

## The Perivascular Niche in Adult Bone

Classical bone marrow derived stromal cells (BMSCs) have been described as fibroblast-like and adherent and capable of forming colonies cells obtained from adult bone marrow (Friedenstein et al., 1970). Similarly, human BMSCs are most often obtained from adult bone marrow (Sacchetti et al., 2007). Accordingly, we first discuss a potential niche for adult BMSCs, referred to here as bone marrow skeletal stem and progenitor cells (bm-SSPCs), both to highlight their predominant characteristics *in vivo* and in alignment with the terminology discussed in section “Historical Perspective on Mesenchymal Stem Cells (MSCs) and Issues of Terminology” above.

Among the various genetic markers utilized to characterize adult bm-SSPCs *in vivo*, the leptin receptor (LepR) is among those studied most extensively, primarily employing transgenic LepR-Cre mice (Ding et al., 2012; Zhou B.O. et al., 2014; Tikhonova et al., 2019). LepR-traced cells contribute little to bone formation during the early postnatal period, but as mice age, most osteoblasts and osteocytes are derived from LepR+ cells (Zhou B.O. et al., 2014). The progeny of LepR+ cells also differentiate into bone marrow adipocytes, but not into chondrocytes of the growth plate under normal physiological conditions. However, during fracture healing the progeny of LepR+ cells constitute almost half of the chondrocytes in the callus (Zhou B.O. et al., 2014). LepR+ cells also express markers for bm-SSPCs such as Prx1 (*Prrx1)*, PDGFRα (*Pdgfra*) and CD51 (*Itgav)* and can form colonies and differentiate into cells of three different lineages *in vitro* (Zhou B.O. et al., 2014). At the same time, the population of cells marked by LepR-Cre is heterogeneous, consisting of two adipocyte-bias and two osteoblast-bias subpopulations, of which it is mainly the former that have the ability to form colonies (Tikhonova et al., 2019).

LepR+ bm-SSPCs are localized in close proximity to the sinusoids and arterioles of the bone marrow (Ding et al., 2012; Zhou B.O. et al., 2014). Therefore, it seems likely that endothelial cells and circulating hormones participate in the formation and/or regulation of the stem cell niche for LepR+ bm-SSPCs. Indeed, it has been proposed that leptin regulates the balance between the osteo- and adipogenic differentiation of bm-SSPCs (Yue et al., 2016). Specifically, activation of Janus kinase 2 (Jak2), an intracellular downstream effector of LepR, in the mesenchyme of the limb bud (in Prx1-Cre:Jak2V617F mice) reduces the trabecular bone mass and increases the number of adipocytes (Yue et al., 2016). Ablation of LepR using Prx1-Cre enhances the expression of markers of osteogenesis (i.e., *Wnt4*), while attenuating the levels of markers of adipogenesis (i.e., *Socs3* and *Cebra*) (Yue et al., 2016). However, these observations must be interpreted with care, since non-inducible Prx1-Cre is active at the limb bud stage (from embryonic day (E) 9.5) and when employed for genetic alterations will induce them in all cells of mesenchymal origin in the limb, including stromal cells, chondrocytes, osteoblasts, osteocytes, and adipocytes (Logan et al., 2002). Thus, the phenotypes observed may be generated indirectly.

It is also unclear whether systemic leptin or leptin secreted by adipocytes in the bone marrow is involved in the regulation of LepR+ bm-SSPCs (Fellows et al., 2016). Adipocytes derived from a CD45-CD31-Sca1+CD24+ mesenchymal-like population of cells, which also express LepR and Cxcl12, secrete dipeptidyl peptidase-4 (Dpp4), which promotes the adipogenic differentiation of CD45-CD31-Sca1+CD24+ cells, forming a positive feedback loop (Ambrosi et al., 2017). *In vitro* sitagliptin, an inhibitor of Dpp4 used clinically to treat diabetes mellitus type 2, promotes osteogenesis, but does not affect the angiogenesis of either CD45-CD31-Sca1+CD24+ cells or CD45-CD31-Sca1-PDGFRα+ osteochondroprogenitors. *In vivo* both sitagliptin and another inhibitor of Dpp4, the tripeptide diprotin A, raise the number of osteochondroprogenitors, lower the number of adipocytes, and promote fracture healing (Ambrosi et al., 2017). On the other hand, single-cell RNA sequencing (scRNAseq) of cells traced with either LepR or vascular endothelial cadherin (VE-Cadherin, also known as CD144) revealed that they do not express *Dpp4* (Tikhonova et al., 2019). Thus, the nature of the Dpp4-dependent interaction between adipocytes and LepR+ bm-SSPCs requires further clarification. Since Dpp4 cleaves a number of chemokines and cytokines, including SDF1 (Zhong and Rajagopalan, 2015), the role of this peptidase in controlling the behavior of stem cells may turn out to be quite complex.

As mentioned above, the close proximity of LepR+ bm-SSPCs to blood vessels suggests that endothelial cells can contribute to the SSPCs niche, as they in fact do to the HSC niche (Winkler et al., 2012; Itkin et al., 2016). Indeed, LepR+ cells express both PDGFRα (*Pdgfra*) and PDGFRβ (*Pdgfrb*) (Sugiyama et al., 2006; Zhou B.O. et al., 2014), whose ligands PDGF-B and PDGF-D are produced by endothelial cells (and pre-osteoclasts) (Xie et al., 2014; Böhm et al., 2019). The existence of such crosstalk is supported by the recent finding that ablation of PDGFRβ from Osx-Cre-positive cells has no effect on bone formation, but lowers the contribution of Osx-traced cells to fracture healing and impaired vascularization of the callus (Böhm et al., 2019).

In addition, endothelial cells and LepR+ bm-SSPCs may interact via the Notch and BMP pathways. Endothelial cells express the Notch ligands *Dll4* and *Dll1*, while LepR+ cells express the *Notch3* receptor (Tikhonova et al., 2019). In addition, LepR+ cells secrete BMP4, while endothelial cells express the *Bmpr2* receptor (Tikhonova et al., 2019). Altogether, these observations suggest that extensive interactions between endothelial cells and bm-SSPCs may contribute to the niche microenvironment.

Of the intracellular signaling pathways likely to be involved in the regulation of LepR+ bm-SSPCs, PI3K/Akt/mTORC1 signaling appears to be a highly plausible candidate. Ablation of the phosphatase and tensin homolog (Pten) in LepR+ cells forces them to differentiate toward the adipogenic lineage (Zhou B.O. et al., 2014). Since Pten counteracts the activity of PI3K, this finding suggests that endocrine/paracrine modulators of the PI3K/Akt/mTORC1 pathway, such as insulin and insulin-like growth factors, may participate in creating a niche appropriate for bm-SSPCs.

Healing of fractures begins in an inflammatory environment. Interestingly, chronic inflammation connected to aging reduces the number of LepR+ bm-SSPCs, as well as their capacity to participate in repair; whereas blocking chronic inflammation with non-steroidal anti-inflammatory drugs (NSAIDs) improves bone regeneration and elevates the number of bm-SSPCs (Josephson et al., 2019). These effects probably involve inhibition of the nuclear factor kappa-light-chain-enhancer in activated B cells (NF-κB), which is otherwise stimulated by SASP (senescence-associated secretory phenotype) (Josephson et al., 2019).

Several other intracellular factors appear to influence the functionality of bm-SSPCs. For instance, ablation of Hox11 (T-cell leukemia homeobox protein 1, *Tlx1* gene) impairs differentiation of bm-SSPCs into cells of the chondrogenic lineage during repair of fractured zeugopod bones, compromising healing or abrogating this process entirely (Rux et al., 2016, 2017). Specific ablation of the forkhead box C1 gene *Foxc1* in LepR+ cells and their progeny (employing LepR-Cre mouse strain) increases the number of adipocytes in the bone marrow, suggesting that *Foxc1* inhibits the adipogenic differentiation of bm-SSPCs (Omatsu et al., 2014). Finally, deletion of the Ebf1 and Ebf3 transcription factors in LepR-targeted cells promotes osteogenesis, suggesting that these factors are required for inhibiting the osteogenic differentiation of bm-SSPCs (Seike et al., 2018).

### Other Genetic Markers and Strains of Mice Employed to Characterize Adult bm-SSPCs

All four subpopulations of LepR+ cells express high levels of *Cxcl12* (Tikhonova et al., 2019). The reciprocal analysis of cells targeted in Cxcl12-CreERT mice reported recently (Matsushita et al., 2020) demonstrated that under normal physiological conditions the labeled cells express LepR strongly and are relatively quiescent, contributing predominantly to adipogenesis and only negligibly to the generation of trabecular osteoblasts (Matsushita et al., 2020). This is in contrast to LepR+ bm-SSPCs that produce as much as 90% of the cells of the osteo-lineage with age (Zhou B.O. et al., 2014), a difference that may reflect the fact that in transgenic Cxcl12-CreERT mice only a subset of Cxcl12+ cells is labeled genetically (Matsushita et al., 2020).

Interestingly, in connection with perturbations such as drilling into the bone or the healing of transverse fractures in the tibia, Cxcl12-traced cells contribute a large proportion of the chondrocytes in the fracture callus, as well as the vast majority of the osteoblasts/osteocytes in the formed cortical bone. The selective ablation of β-catenin in this Cxcl12-positive population of cells impairs their osteoblastic differentiation and ability to participate in fracture healing (Matsushita et al., 2020). Thus, in theory at least, bm-SSPCs labeled with Cxcl12-CreERT may represent a subpopulation of LepR+ cells that is activated specifically in response to pathological conditions and β-catenin pathway is involved either in their activation or subsequent functions.

LepR+ bm-SSPCs also express Prx1 (Zhou B.O. et al., 2014, Tikhonova et al., 2019) and activation of hedgehog signaling in Prx1+ cells in 2-week-old Prx1-CreERT mice (Kawanami et al., 2009) promotes their osteogenic differentiation, both *in vivo* and *in vitro* (Deng et al., 2019). Thus, hedgehog signaling may be required for the proper functioning of bm-SSPCs.

In addition to the use LepR as a genetic marker, the bm-SSPCs of adult mice can also be labeled on the basis of their expression of *Grem1*, utilizing the inducible Grem1-CreERT mouse strain (Worthley et al., 2015). Although the populations of LepR+ and Grem1+ bm-SSPCs do not necessarily overlap, scRNAseq analysis revealed expression of *Grem1* by the peri-sinusoidal fraction of LepR-traced cells destined preferentially to become adipocytes (Tikhonova et al., 2019). At the same time, Grem1+ cells in adult mice are predominantly osteogenic (Worthley et al., 2015), suggesting that there exists a subset of Grem1+ cells distinct from the LepR+ population. Grem1+ cells in adult mice are characterized by high expression of *Bmp2*, *Bmp5*, and *Bmp6*, as well as pronounced BMP signaling (Worthley et al., 2015) that may be involved in regulating bm-SSPCs. It is noteworthy that the KEGG (Kyoto Encyclopedia of Genes and Genomes) analysis shows high levels of activities for the extracellular matrix-receptor interaction, PI3K/Akt/mTORC1 and focal adhesion pathways in Grem1+ cells (Worthley et al., 2015).

Another marker utilized to identify adult bm-SSPCs is the glioma-associated oncogene homolog zinc finger protein 1 (Gli1) (Shi et al., 2017). Adult Gli1+ cells characterized employing inducible Gli1-CreERT:tdTomato mice, when labeled at one month of age generate osteoblasts and bone marrow adipocytes with time and, if analyzed 24 h after the labeling, about 60% of these cells are found to express PDGFRα and 10% LepR (Shi et al., 2017). However, 5 months later already 50% of the Tomato+ cells express LepR (Shi et al., 2017). It remains to be determined whether this finding reflects a hierarchical relationship between Gli1+ and LepR+ bm-SSPCs or age-dependent expression of LepR.

Ablation of the HH pathway in adult Gli1+ cells impairs their osteogenic differentiation (Shi et al., 2017), in good agreement with the observation (described in more detail above) that activation of this pathway promotes the osteogenic differentiation of Prx1+ bm-SSPCs (Deng et al., 2019). Some evidences also indicate that β-catenin-dependent Wnt signaling is involved in the regulation of adult bm-SSPCs. Specifically, inactivation of β-catenin in adult Gli1+ cells favors their adipogenic differentiation (Shi et al., 2017). This aligns well with the previous observation that Osx-Cre mediated ablation of β-catenin causes a shift of the fate of targeted cells to the adipogenic line (Song et al., 2012). However, in this context it should not be forgotten that Osx-Cre targets a number of different types of cells, including osteoprogenitors, pericytes and bm-SSPCs (Mizoguchi et al., 2014; Böhm et al., 2019).

These observations indicate that the HH, BMP, Wnt, and mTORC1 pathways are all involved in determining the fate of adult bm-SSPCs and, accordingly, external effectors of these pathways may be involved in creating a niche designed for bm-SSPCs.

### Characterization of bm-SSPCs Using the Nestin-GFP and Non-inducible Prx1-Cre Mouse Strains

The Nestin-GFP and non-inducible Prx1-Cre mouse strains are often used to characterize adult bm-SSPCs but have some limitations. An early, highly influential article identified Nestin (*Nes*) as a key marker of bm-SSPCs (referred to as MSCs in that publication), largely on the basis of analysis of Nestin-GFP transgenic mice (Méndez-Ferrer et al., 2010). However, later investigations revealed that the expression of transgenic Nestin-GFP does not reflect the expression of endogenous Nestin or of the transgenic Nestin-Cre construct (Ding et al., 2012; Zhou B.O. et al., 2014), which complicates interpretation of numerous observations considerably. Employing another Nestin-CreERT strain, Worthley et al. (2015) were able to target only 4% of the Nestin-GFP cells, which revealed no contribution to the osteoblast lineage. Furthermore, LepR+ bm-SSPCs do not express the *Nes* gene (Zhou B.O. et al., 2014, Tikhonova et al., 2019) and the population of Grem1+ cells is distinct from that of Nestin-GFP cells (Worthley et al., 2015). At the same time, other investigators have reported detection of low levels of Nestin-GFP in LepR-targeted cells, so called Nestin-GFP^dim^ cells, located close to sinusoids (Ding et al., 2012; Kunisaki et al., 2013). Moreover, 87% of sorted adult Nestin-GFP^high^ cells have been reported to express *Lepr* (Li et al., 2016). Thus, there is a considerable confusion in the scientific literature concerning the use of Nestin-GFP as a marker for bm-SSPCs cells. To complicate this situation even more, the limited overlap between the Nestin-GFP and Nestin-CreERT transgenic strains of mice (Worthley et al., 2015) makes it difficult to use these as model systems to explore the contribution of Nestin-GFP^high^ cells to the generation of adipocytes, chondrocytes and osteoblasts *in vivo*.

In the case of Prx1-Cre mice, it has been reported that in 10-week-old animals LepR+ cells express *Prx1* (Zhou B.O. et al., 2014, Tikhonova et al., 2019) and 87–89% of the Prx1-Cre-traced cells express LepR in adult mice (Yue et al., 2016). On other hand, cells of the Prx1-Cre-traced subpopulation within the PDGFRα+Sca1+lin-CD45 cells form colonies, but do not express *Nes* or *Lepr* (Greenbaum et al., 2013). Thus, it seems likely that the Prx1-traced cells comprise bm-SSPCs, including LepR+ bm-SSPCs. However, as mentioned above, it must be remembered in this context that the non-inducible Prx1-Cre causes recombination in the limb bud mesenchyme from day E9.5 onward (Logan et al., 2002).

Nevertheless, numerous interesting observations are made with Nestin-GFP and non-inducible Prx1-Cre mice and can shed light on the composition of the adult bm-SSPCs niche and will, therefore, be discussed below.

Sympathetic nerve fibers connected to perivascular stromal cells regulate Nestin-GFP cells. More specifically, chemical neurectomy increases the proliferative activity of Nestin-GFP^high^ cells, whereas treatment with β3 adrenergic receptor agonists inhibits the osteogenic differentiation of these same cells in culture (Méndez-Ferrer et al., 2010). Expression of the gap junction proteins gamma 1 (connexin-45, encoded by *Gjc1*) and alpha 1 (connexin 43, encoded by *Gja1*) by Nestin-GFP^high^ cells suggest that they may have an electromechanical connection to noradrenergic nerve fibers (Méndez-Ferrer et al., 2010). In addition, with aging the bone marrow becomes less innervated and the number of Nestin-GFP^high^ cells, but not Nestin-GFP^dim^ cells rises (Maryanovich et al., 2018).

Furthermore, a functional sympathetic nervous system is required for the anti-osteogenic action of leptin (Takeda et al., 2002). In another model, elevated production of IL-1β by HSCs due to a constitutive mutation in Jak2 (mutation *p.V617F*) results in neuronal damage and decreases the number of Schwann cells, which in turn causes the death of Nestin-GFP^dim^ cells; and this effect is attenuated by an antagonist of β3-adrenoceptors, BRL37344 (Arranz et al., 2014). In this connection it is worth mentioning that sympathetic nerve fibers are involved in regulating a niche for HSCs through secretion of norepinephrine (Katayama et al., 2006), as well as in regulation of the stem cell niche in hair follicles via secretion of Shh (Brownell et al., 2011). This apparent involvement of sympathetic nerve fibers in the bm-SSPCs niche certainly warrants further investigation.

Intermittent treatment of bone with parathyroid hormone (PTH) or PTH-related peptide (PTHrP) exerts a well-known anabolic effect (Osagie-Clouard et al., 2017). After PTH treatment of mice, Nestin-GFP^high^ cells isolated from these animals demonstrate enhanced proliferation and differentiation into osteoblasts in culture (Méndez-Ferrer et al., 2010; Ding et al., 2012). Genetic ablation of the PTH/PTHrP Receptor (*Pth1r*) in Prx1-targeted cells enhances bone adiposity, whereas treatment of isolated bm-SSPCs with PTH1-34 promotes osteogenic differentiation (Fan et al., 2017). The chemokine Cxcl12, which is secreted in considerable amounts by stromal and mesenchymal cells and is known play a role in maintaining the HSC niche (Sugiyama et al., 2006), appears to be involved in supporting the bm-SSPCs niche as well. Targeted deletion of *Cxcl12* in Prx1-targeted cells reduces the mass and elevates the adiposity of bone (Tzeng et al., 2018). Since Prx1-specific ablation of the Cxcl12 receptor Cxcr4 (*Cxcr4*) reduces bone mass without affecting marrow adiposity, it remains unclear whether the effect is autonomous for bm-SSPCs (Tzeng et al., 2018).

Granulocyte colony-stimulating factor (G-CSF or Csf3) secreted by monocytes, fibroblasts and endothelial cells inhibits both the expansion of Nestin-GFP^high^ cells and their differentiation toward the osteoblast lineage (Méndez-Ferrer et al., 2010).

The receptor activator of nuclear factor kappa-B ligand (RANKL) is secreted not only by osteoblasts, but also by bm-SSPCs (Fan et al., 2017; Chen et al., 2018). In addition to regulating osteoclastogenesis (Lacey et al., 1998), RANKL attenuates the differentiation of bm-SSPCs into cells of the osteogenic lineage (Cao, 2018; Chen et al., 2018). This inhibition is probably mediated by activation of NF-κB, with subsequent inhibition of Wnt/β-catenin signaling (Chen et al., 2018).

Furthermore, both osteocytes and osteoblasts secrete osteoprotegerin (OPG), a soluble decoy receptor for RANKL (Kramer et al., 2010), and it appears plausible that a balance between RANKL and OPG may regulate the behavior of bm-SSPCs, creating a feedback signal. Another regulatory link between bm-SSPCs and their progeny may involve Semaphorin 3A (Sema3a), which is secreted by osteoblasts and osteocytes and may promote osteogenic differentiation of bm-SSPCs (Hayashi et al., 2012; Qiao et al., 2018). Altogether, PTH, Cxcl12, G-CSF, the RANKL/OPG ratio and Sema3a may potentially contribute to the formation and regulation of the bm-SSPCs niche in an endocrine/paracrine/autocrine fashion. Further research will establish precise roles played by these different factors.

Clearly, the extracellular matrix plays a role in the regulation of bm-SSPCs *in vitro* (Hoshiba et al., 2016), as well as in the regulation of other stem cell niches *in vivo* (Gattazzo et al., 2014). However, *in vivo* evidence for an involvement of the extracellular matrix in the regulation of adult bm-SSPCs is rather limited. Overexpression of dentin matrix protein 1 (DMP1) under the transcriptional control of the *Nes* promoter attenuates the proliferation and osteogenic differentiation of Nestin+ cells, leading subsequently to a reduction in bone mass (Pan et al., 2017). In contrast, Prx1-dependent ablation of *Dmp1* enhances bone mass and the number of osteoblasts *in vivo* and promotes the *ex vivo* osteogenic differentiation of cells expressing Prx1 (Zhang et al., 2018). Thus, DMP1 may be one of the components of the extracellular matrix involved in niche formation.

In this context it is worth noting that DMP1 is secreted primarily by the progeny of bm-SSPCs, i.e., late osteoblasts and osteocytes (Fan et al., 2017; Chen et al., 2018). Interestingly concerning the extracellular matrix, a classic test for bone formation involving injection of bm-SSPCs under the kidney capsule shows that these cells can form bone only when co-injected either with their progeny (Chan et al., 2015) or matrigel (Debnath et al., 2018), the extracellular matrix of mouse sarcoma. Thus, their progeny and/or the extracellular matrix are key components of the niche required for maintenance of bm-SSPCs.

In summary, our understanding of the adult bm-SSPCs niche is still evolving. Key components of this niche probably include their immediate progeny, the progeny of HSCs (e.g., monocytes and osteoclasts), adjoining endothelial cells, sympathetic nerve fibers, the extracellular matrix and various hormones coming both from the bloodstream, as well as acting in a paracrine manner (Figure 1D).

## Skeletal Stem and Progenitor Cells in Developing Bone

Different genetic markers and/or various other approaches have been utilized to characterize several populations of skeletal stem cells in fetal, neonatal or early postnatal bones. Some of these have already been mentioned above (e.g., Prx1+, Gli1+, Grem1+, Nestin+) and others include Sox9+ SSPCs (Akiyama et al., 2005; He et al., 2017), Col2+ SSPCs (Ono et al., 2014b), Osx+ SSPCs (Greenbaum et al., 2013; Mizoguchi et al., 2014; Tzeng et al., 2018) and Lin-AlphaV+CD200+ SSPCs (Short et al., 2009). Genetic labeling reveals that some of these give rise to others during development (i.e., cells expressing Prx1 begin expressing Osx, Sox9, and Col2), while other populations overlap partially (i.e., Sox9+ and Col2+ cells, Osx+ and Prx1+ cells). In addition, essentially every mouse strain mentioned above targets perichondrium (i.e., Prx1, Nestin-GFP, Sox9, Col2, Grem1, Gli1, and Osx markers) and/or the growth plate chondrocytes (i.e., Prx1, Sox9, Col2, Gremlin, and Gli1 markers). It is important to emphasize that during development the perichondrium gives rise to bone marrow stroma (Maes et al., 2010) and, as discussed above, retains SSPCs into adulthood (Yang et al., 2013; Debnath et al., 2018). Moreover, *trans*-differentiation of hypertrophic chondrocytes of the growth plate into various mesenchymal-type cells of the bone marrow is well established (Yang G. et al., 2014, Yang L. et al., 2014; Zhou X. et al., 2014) and particularly intensive during the early stages of longitudinal bone growth (Li et al., 2017; Mizuhashi et al., 2018; Newton et al., 2019).

The potential influx of new stem/progeny cells from other sources [e.g., Schwann and endoneurial fibroblasts (Carr et al., 2019; Xie et al., 2019b)] makes the development of the skeleton even more complex. From our perspective, the information presently available is indicative of phenotypic plasticity of cells of mesenchymal original and high developmental dynamics during neonatal growth. FACS-based characterization of SSPCs such as Lin-AlphaV+CD200+ SSPCs (Chan et al., 2015, 2018) obtained from surgical samples of fetal or neonatal growth plate surrounded by innervated perichondrium may resolve this issue by identifying *bona fide* skeletal stem cells. However, this approach does not reveal their exact location or the nature of their microenvironment and allows only limited manipulation of these cells in their natural *milieu*.

Of course, every population of SSPCs identified provides valuable information, but, at the same time, little insight can be made into location or composition of the niche during this period. Indeed, *in vivo* identification and localization of the progeny of any specific type of SSPCs is virtually impossible in this dynamic setting. Nevertheless, one pattern is becoming clear.

Several markers – including Grem1 (Worthley et al., 2015), Gli1 (Shi et al., 2017), LepR (Zhou B.O. et al., 2014), Osx (at its multipotency stage, Mizoguchi et al., 2014), PDGFRβ (Böhm et al., 2019), Prx1 (Greenbaum et al., 2013), Nestin-GFP (Ono et al., 2014a), and Col2 (Ono et al., 2014b) – reveal the presence of putative stem cells in the region of the primary spongiosa, immediately below the growth plate. The primary spongiosa is a unique area characterized by intensive bone formation and tissue remodeling. Its distinct extracellular matrix is comprised of remnants of calcified cartilage enriched in type X collagen and osteopontin and containing high levels of matrix metalloproteinases such as MMP9, MMP13, and MMP14, highly active osteoclasts, active *trans*-differentiation of hypertrophic chondrocytes, active angiogenesis and unique arrangements of endothelial cells into hemospheres (Wang et al., 2013).

Furthermore, hypertrophic chondrocytes express a variety of cytokines, including vascular endothelial growth factor (VEGF), RANKL, OPG, and Ihh (Houben et al., 2016), which also may participate in creating a proper microenvironment for SSPCs. For example, ablation of *Ihh* in the growth plate employing Col2-Cre attenuates both Wnt signaling and the maturation of osteoblasts within the primary spongiosa (Maeda et al., 2007). Thus, this combination of features may be the key to creating and maintaining the SSPC niche in growing bones.

However, detailed determination of the components of this niche and their roles in the regulation of individual populations of SSPCs within the primary spongiosa will probably require approaches that are more advanced and sophisticated than those been utilized to date. Moreover, it is important to remember that in humans the growth plate fuses (disappears) during late puberty, in association with the cessation of growth, whereas in mice the growth plate remains open while these animals continue growing into adulthood (Emons et al., 2011; Chagin and Newton, 2019). Accordingly, findings on mice must be applied to humans only with care.

## Conclusion

Our present knowledge concerning stem cell niches comes mainly from studies on the epithelial and hematopoietic niches, while stem cell niches for SSPCs have been characterized much less extensively. Above, we summarize known information and our own current ideas about the composition of such niches within bones and the key regulatory pathways that may be involved in establishing and maintaining them.

Each stem cell niche is a complex microenvironment, with an influx of converging signals that influence the behavior of stem cells. We propose that these signals can be categorized as either primary or modulating, with the former being responsible for maintaining basic functions, such as the renewal of stem cells and generation of their progeny, whereas the latter adapt stem cell behavior to changing conditions. Identification of these signals associated with niches for mesenchymal-type cells could be of considerable value in connection with various regenerative therapeutic approaches.

In summary, all of the skeletal stem cell niches discussed here have three features in common: (i) feedback from committed or differentiated progeny, (ii) interaction with the extracellular matrix, and (iii) responsiveness to mechanical and chemical stimuli. Moreover, hedgehog and Wnt signaling, along with inflammatory signals appear to be most common regulators for niches of mesenchymal-type cells (see also Figure 1). Clearly, a better understanding of the microenvironments provided by such niches can suggest novel therapeutic approaches based on the regulation of SSPCs.

## Author Contributions

AC and AK conceived the study. All authors contributed to reviewing the literature, intellectual discussion of the findings, and drafting the manuscript.

## Conflict of Interest

The authors declare that the research was conducted in the absence of any commercial or financial relationships that could be construed as a potential conflict of interest.
